# Supplementary material for: Genomic C-Value Variation Analysis in Jujube (Ziziphus jujuba Mill.) in the Middle Yellow River Basin
Source: Plants (Basel). 2023 Feb 14;12(4):858. doi: 10.3390/plants12040858 (PMC9962250; doi:10.3390/plants12040858)
Supplement: Supplementary file 1 [file plants-12-00858-s001.zip › plants-2037022-supplementary/S1-S4/Table S2.pdf]

Table S2 Correlation analyses between genome size and multiple fruit traits.

Trait	SRW	FLD	FTP	SW	SF	TS	TA	VC	FER	GS
SRW	1.00	0.53***	0.68***	0.40***	-0.23	-0.01	-0.21**	-0.16	0.09	**0.25***
FLD		1.00	-0.45***	0.19	-0.04	0.07	0.15	0.15	0.50***	-0.04
FTP			1.00	0.20	0.18	0.02	0.19	0.02	0.33***	0.02
SW				1.00	-0.07	-0.11	-0.15	-0.03	-0.79***	-0.08
SF					1.00	0.52***	-0.04	0.06	-0.15	-0.10
TS						1.00	-0.05	-0.06	-0.01	0.06
TA							1.00	0.16	-0.28*	0.15
VC								1.00	0.07	**0.38*****
FER									1.00	-0.05
GS										1.00

NOTE: Single star * means that r value was significant at the level of 0.01. Single star ** means that r value was significant at the level of 0.001 Single star *** means that r value was significant at the level of 0.0001. SRW: Single fruit weight; FLD: Fruit length; FTP: Fruit width; SW: Stone weight; SF: Soluble solids content; TS: Total soluble sugar content; TA: Titratable acid content; VC: Vitamin C content; FER: fresh edible rate; GS: genomic C value
