# Supplementary material for: Genomic C-Value Variation Analysis in Jujube (Ziziphus jujuba Mill.) in the Middle Yellow River Basin
Source: Plants (Basel). 2023 Feb 14;12(4):858. doi: 10.3390/plants12040858 (PMC9962250; doi:10.3390/plants12040858)
Supplement: Supplementary file 1 [file plants-12-00858-s001.zip › plants-2037022-supplementary/S1-S4/Table S3.pdf]

Table S3 Fruit trait data of 89 representative jujube germplasm

No. 1	Germplasm name	SRW g	FL D cm	FTP cm	SW g	SF %	TS %	TA %	VC mg/(100g)	FER %	ploidy	genome size/Mb
1	LXMJW-1	18.1	4.22	3.10	0.42	28.67	26.08	0.82	527.04	97.66	2X	499.93
2	LXMJW-2	11.3	3.83	2.54	0.32	32.00	30.87	0.56	410.68	97.20	2X	482.39
3	LXMJW-3	15.2	4.78	2.67	0.45	28.33	29.49	0.85	468.97	97.08	2X	471.76
4	LXMJW-4	21.1	4.16	3.57	0.45	28.33	29.49	0.85	468.97	97.87	2X	491.65
5	LXXZZ-1	11.8	3.97	2.61	0.29	27.00	25.67	0.81	509.09	97.55	2X	490.61
6	LXWJZDZ	14.9	3.97	2.88	0.29	27.00	25.48	0.52	455.45	98.08	2X	488.4
7	JSCZ	13.8	3.98	2.80	0.26	28.00	25.69	0.68	455.90	98.10	2X	503.06
8	LXYJZ-2	17.9	4.59	3.06	0.49	27.00	26.53	0.36	454.34	97.28	2X	465.14
9	YHTZ	10.6	4.04	2.37	0.26	30.67	30.36	0.60	496.45	97.54	2X	469.26
10	LXWJZMYZ	6.5	3.00	2.24	0.27	27.00	25.48	0.52	455.45	95.80	2X	444.6
11	LXQYNNZ	13.5	4.76	2.45	0.35	29.00	25.43	0.60	487.05	97.40	2X	423.14
12	LXXZZ-2	9.1	3.07	2.52	0.28	29.50	26.58	0.46	324.50	96.87	2X	427.22
13	LXYZCZ	13.2	3.46	2.80	0.75	27.67	27.04	1.35	402.93	94.30	2X	428.09
14	LXKYDSZ	7.0	2.68	2.31	0.48	26.50	19.22	1.73	579.91	93.15	2X	467.64
15	LXEBCCMZ	10.1	3.63	2.46	0.20	29.00	24.58	0.63	467.32	97.97	2X	450.82
16	LXLJHPBB	11.9	3.70	2.57	0.41	32.50	28.08	0.86	312.66	96.58	2X	473.42
17	LXLJHZ	13.2	4.34	2.64	0.32	26.00	20.38	1.03	671.88	97.58	2X	449.53
18	LXQKNXC	12.5	3.90	2.65	0.34	33.00	28.68	0.60	460.11	97.30	2X	415.07
19	LXQKBZ	12.9	4.26	2.73	0.39	29.0	26.1	0.6	373.3	97.01	2X	419.37
20	LXYLLZ	16.4	4.32	2.96	0.71	31.50	32.31	0.60	373.21	95.67	2X	402.58
21	LXLJPAAZ	20.7	4.74	3.33	0.53	30.00	21.07	0.38	554.56	97.45	2X	480.61
22	PYBJZ	15.4	3.98	2.96	0.48	26.00	21.24	0.40	301.69	96.91	2X	441.26
23	YCZCGYZ	10.0	3.22	2.53	0.43	33.00	25.46	0.43	395.35	95.67	2X	411.46
24	YCZCTSZ	7.1	2.98	2.27	0.47	29.00	23.21	0.82	289.80	93.35	2X	416.77
25	YCZZXSYZ	9.6	3.39	2.53	0.43	26.00	23.42	0.65	307.92	95.52	2X	398.2
26	YCLYDSZ	10.2	4.19	2.33	0.35	29.67	25.55	0.61	425.31	96.62	2X	410.92
27	DLMGX-1	9.0	3.11	2.39	0.53	32.50	27.18	0.42	217.86	94.07	2X	434.52
28	LXYJMYZ	14.6	4.04	2.82	0.37	33.50	29.65	0.71	281.12	97.50	2X	452.83
29	XXLPZ	15.2	3.75	2.95	0.43	27.00	26.20	0.42	510.69	97.15	2X	439.37
30	LXQJSLJZ	8.9	3.90	2.44	0.35	30.00	28.42	0.39	444.85	96.13	2X	424.5
31	LXQJSDMZ	12.1	4.15	2.60	0.32	30.00	28.42	0.39	444.85	97.39	2X	429.05
32	LXQJS-1	10.8	3.88	2.50	0.34	31.00	28.42	0.51	467.35	96.84	2X	437.73
33	YCCMZ	14.0	3.96	2.76	0.40	29.50	27.47	0.52	430.41	97.15	2X	452.57
34	QXMGZ	19.8	3.82	3.29	0.63	28.00	26.29	0.58	208.65	96.85	2X	438.22
35	JSSZ	17.0	4.13	3.15	0.63	28.00	22.70	0.68	195.76	96.28	2X	443.36
36	JSHMZ	7.9	2.93	2.33	0.24	35.00	28.88	0.55	366.58	96.97	2X	434.33
37	JSZYZ	13.0	3.75	2.75	0.32	29.50	26.58	0.46	324.50	97.58	2X	451.37
38	YCMZ	9.1	3.37	2.47	0.23	30.00	27.40	0.67	297.00	97.53	2X	429.37
39	LXSEZ	7.4	3.31	2.27	0.31	31.00	27.75	0.61	514.10	95.83	2X	434.98
40	LXDSZ	4.9	2.32	2.03	0.50	27.67	27.04	1.35	402.93	89.79	2X	434.52

41	LXHZ	26.5	4.89	3.50	0.78	34.00	25.53	0.39	259.39	97.04	2X	440.28
42	LYHDZ	9.0	4.10	2.16	0.31	27.00	24.97	0.67	246.68	96.63	2X	410.01
43	LYDCGZ	12.8	4.63	2.42	0.48	29.0	26.1	0.6	373.3	96.24	2X	425.29
44	LFSZ	8.0	3.46	2.80	0.75	27.67	27.04	1.35	402.93	94.30	2X	441.8
45	YCDBZ	23.1	3.53	3.21	0.92	24.00	23.04	0.16	221.58	96.00	2X	425.07
46	YCBZ	13.2	3.55	2.85	0.87	29.40	29.08	0.29	382.16	93.42	2X	424.64
47	LYLZ	31.6	4.54	3.96	1.08	27.00	22.50	0.37	292.25	95.44	2X	477.47
48	LYLJZ	20.8	4.41	2.91	1.37	27.67	19.25	0.36	288.68	93.40	2X	438.7
49	BDXZ	10.6	3.48	2.57	0.33	33.60	27.36	1.02	415.13	97.60	2X	469.91
50	BDYZ	10.9	3.42	2.66	0.38	33.60	26.65	0.78	511.44	97.22	2X	494.76
51	ZYMZ	10.2	3.75	2.41	0.37	25.20	22.69	0.74	502.90	96.35	2X	460.62
52	ZYTZ	18.3	3.51	3.32	0.57	28.80	24.60	0.41	382.16	96.89	2X	432.6
53	QXYZ	14.6	3.12	3.10	0.68	33.00	24.76	0.56	264.95	93.48	2X	433.51
54	XFYZ	16.4	3.55	2.93	0.62	30.00	24.46	0.48	341.39	96.10	2X	453.21
55	YCPPZ	14.1	3.51	3.09	0.62	30.60	29.00	0.37	489.40	94.53	2X	484.23
56	JCJZ	26.3	5.07	3.46	0.97	33.00	28.68	0.45	432.00	97.00	2X	437.35
57	YJHMZ	25.4	4.93	3.61	0.94	32.40	28.18	0.50	420.82	95.74	2X	558.91
58	XFGTZ	12.6	3.52	2.73	0.59	34.50	24.62	0.39	445.90	95.70	2X	454.82
59	XFMZ	17.9	3.54	2.66	0.52	33.60	31.60	0.59	423.70	94.10	2X	453.22
60	JSLGZ	21.4	4.33	3.44	0.82	33.00	29.48	0.58	397.31	94.78	2X	462
61	LXYJZ-1	11.7	3.93	2.65	0.51	27.90	24.22	0.76	541.06	95.65	2X	495.13
62	PLTTZ	15.7	2.99	3.34	0.58	32.40	22.95	0.50	359.47	95.34	2X	446.88
63	PLJZ	10.2	3.74	2.36	0.56	28.50	21.18	0.52	407.97	94.31	2X	446.17
64	XFYZZ	10.5	3.27	2.56	0.38	27.90	23.27	0.99	499.75	96.38	2X	447.71
65	XXYCZ	9.6	3.01	2.46	0.55	32.40	27.67	0.65	343.61	93.22	2X	452.82
66	XXZYZ	16.2	3.18	3.32	0.68	40.20	30.20	0.79	371.98	94.34	2X	447.59
67	JCDZ	12.5	2.87	2.92	0.82	33.90	22.60	0.65	429.50	92.40	2X	452.83
68	YCDYZ	17.4	3.31	2.81	1.01	22.00	21.94	0.26	429.50	94.20	2X	435.16
69	DLXDD	7.1	2.40	2.30	0.54	33.60	26.73	0.66	334.02	92.65	2X	430.5
70	DLMYZ	24.0	4.22	2.55	1.00	28.60	24.76	0.50	245.29	92.53	2X	469.09
71	DLJDZ	17.2	3.97	3.14	0.83	27.00	23.50	0.43	266.17	96.41	2X	435.92
72	PCMZ	17.3	3.45	3.15	0.99	37.20	28.80	0.57	446.33	92.91	2X	433.12
73	PCYLZ	19.0	3.69	3.24	0.66	36.00	29.92	0.78	426.92	95.96	2X	422.64
74	PCDDZ	19.6	3.84	3.28	0.92	33.60	24.93	0.47	372.29	94.81	2X	425.89
75	DLLZ	11.6	3.73	2.60	0.55	28.80	21.31	0.59	367.88	93.95	2X	439.54
76	DLLLZ	10.6	3.06	2.88	0.54	31.80	27.13	0.60	335.50	93.60	2X	429.49
77	LTGLZ	12.1	3.38	2.76	0.63	31.20	24.12	0.89	431.45	93.80	2X	428.56
78	DLYZ	20.3	3.92	3.37	0.85	34.20	27.74	0.72	196.01	92.89	2X	417.87
79	DLGWB	16.4	3.75	3.12	0.89	32.40	26.35	0.59	209.43	94.57	2X	413.35
80	YCTZ	13.1	4.15	2.50	0.44	36.15	19.42	0.68	467.57	96.64	2X	417.63
81	DLPCZ	11.4	3.43	2.74	0.53	28.20	21.60	0.57	457.60	94.64	2X	420.59
82	DLZZ	14.0	3.66	3.03	0.72	30.90	25.66	0.67	237.62	93.83	2X	421.06
83	DLFMG	9.2	2.70	2.52	0.55	31.80	25.97	0.51	359.05	94.02	2X	429.41
84	DLGDZ	18.8	3.62	3.17	0.94	31.20	24.31	0.52	322.20	93.51	2X	415

85	DLBSH	9.4	2.91	2.38	0.58	25.40	22.39	0.40	312.44	93.80	2X	426.22
86	DLMZ	13.5	3.29	2.78	0.65	36.00	35.74	0.30	447.14	95.19	2X	424.31
87	DLZCBZ	17.9	3.60	3.15	0.75	27.60	22.19	0.51	315.97	94.56	2X	438.85
88	PYKDZ	18.3	3.51	3.47	0.59	30.00	24.86	0.61	378.23	96.92	2X	424.26
89	PYDZ	7.4	3.53	2.26	0.42	29.70	24.20	0.79	447.14	95.35	2X	421.03
