# Supplementary material for: Genomic C-Value Variation Analysis in Jujube (Ziziphus jujuba Mill.) in the Middle Yellow River Basin
Source: Plants (Basel). 2023 Feb 14;12(4):858. doi: 10.3390/plants12040858 (PMC9962250; doi:10.3390/plants12040858)
Supplement: Supplementary file 1 [file plants-12-00858-s001.zip › plants-2037022-supplementary/S1-S4/TableS1.pdf]

Table S1 Information on chromosome number and genomic C value of jujube discussed in this study.

No	Germplasm name	origin	Longitude	Latitude	Genomic C-value		ploidy	genome size/Mb
					Mean±SD(Pg)	CV(%)		
1	LXMJW-1	Linxian,Shanxi	E110°29′40"	N37°57'25"	0.25559±0.00498	1.95	2X	499.93
2	LXMJW-2	Linxian,Shanxi	E110°29′40"	N37°57'25"	0.24662±0.00298	1.21	2X	482.39
3	LXMJW-3	Linxian,Shanxi	E110°29′40"	N37°57'25"	0.24119±0.01001	4.15	2X	471.76
4	LXMJW-4	Linxian,Shanxi	E110°29′40"	N37°57'25"	0.25135±0.00101	0.4	2X	491.65
5	LXXZZ-1	Linxian,Shanxi	E110°29′40"	N37°57'25"	0.25082±0.00800	3.19	2X	490.61
6	LXWJZDZ	Linxian,Shanxi	E110°29′40"	N37°57'25"	0.24969±0.01298	5.2	2X	488.4
7	JSCZ	Jishan,Shanxi	E110°59'21"	N35°36'37"	0.25719±0.00301	1.17	2X	503.06
8	LXYJZ-2	Linxian,Shanxi	E110°29′40"	N37°57'25"	0.23780±0.00100	0.42	2X	465.14
9	YHTZ	Yonghe,Shanxi	E110°38'18"	N36°45'57"	0.23991±0.00499	2.08	2X	469.26
10	LXWJZMYZ	Linxian,Shanxi	E110°29′40"	N37°57'25"	0.22730±0.00200	0.88	2X	444.6
11	LXQYNNZ	Linxian,Shanxi	E110°29′40"	N37°57'25"	0.21633±0.00301	1.39	2X	423.14
12	LXXZZ-2	Linxian,Shanxi	E110°29′40"	N37°57'25"	0.21842±0.00701	3.21	2X	427.22
13	LXYZCZ	Linxian,Shanxi	E110°29′40"	N37°57'25"	0.21886±0.00499	2.28	2X	428.09
14	LXKYDSZ	Linxian,Shanxi	E110°29′40"	N37°57'25"	0.23908±0.00100	0.42	2X	467.64
15	LXEBCCMZ	Linxian,Shanxi	E110°29′40"	N37°57'25"	0.23048±0.00401	1.74	2X	450.82
16	LXLJHPBB	Linxian,Shanxi	E110°29′40"	N37°57'25"	0.24203±0.00600	2.48	2X	473.42
17	LXLJHZ	Linxian,Shanxi	E110°29′40"	N37°57'25"	0.22982±0.00699	3.04	2X	449.53
18	LXQKNXC	Linxian,Shanxi	E110°29′40"	N37°57'25"	0.21220±0.00700	3.3	2X	415.07
19	LXQKBZ	Linxian,Shanxi	E110°29′40"	N37°57'25"	0.2144±0.00101	0.47	2X	419.37
20	LXYLLZ	Linxian,Shanxi	E110°29′40"	N37°57'25"	0.20582±0.00200	0.97	2X	402.58
21	LXLJPAAZ	Linxian,Shanxi	E110°29′40"	N37°57'25"	0.24571±0.00799	3.25	2X	480.61
22	PYBJZ	Pingyao,Shanxi	E112°10'56"	N37°11'44"	0.22559±0.00399	1.77	2X	441.26
23	YCZCGYZ	Yuci,Shanxi	E112°42'52"	N37°42'14"	0.21036±0.00200	0.95	2X	411.46
24	YCZCTSZ	Yuci,Shanxi	E112°42'52"	N37°42'14"	0.21307±0.01099	5.16	2X	416.77
25	YCZZXSYZ	Yuci,Shanxi	E112°42'52"	N37°42'14"	0.20358±0.00599	2.94	2X	398.2
26	YCLYDSZ	Yanchuan,Shaanxi	E110°12'1"	N36°53'2"	0.21008±0.01901	9.05	2X	410.92
27	DLMGX-1	Dali,Shanxi	E109°56'54"	N34°48'6"	0.22215±0.00500	2.25	2X	434.52
28	LXYJMYZ	Linxian,Shanxi	E110°29′40"	N37°57'25"	0.23151±0.00299	1.29	2X	452.83
29	XXLPZ	Xingxian,Shanxi	E111°8'3"	N38°28'7"	0.22463±0.00699	3.11	2X	439.37
30	LXQJSLJZ	Linxian,Shanxi	E110°29′40"	N37°57'25"	0.21702±0.00701	3.23	2X	424.5
31	LXQJSDMZ	Linxian,Shanxi	E110°29′40"	N37°57'25"	0.21935±0.00101	0.46	2X	429.05
32	LXQJS-1	Linxian,Shanxi	E110°29′40"	N37°57'25"	0.22379±0.00600	2.68	2X	437.73
33	YCCMZ	Yanchuan,Shaanxi	E110°12'1"	N36°53'2"	0.23138±0.00010	0.04	2X	452.57
34	QXMGZ	Qingxu,Shanxi	E112°21'52"	N37°36'47"	0.22404±0.00300	1.34	2X	438.22
35	JSSZ	Jishan,Shanxi	E110°59'21"	N35°36'37"	0.22667±0.01000	4.41	2X	443.36
36	JSHMZ	Jishan,Shanxi	E110°59'21"	N35°36'37"	0.22205±0.00300	1.35	2X	434.33
37	JSZYZ	Jishan,Shanxi	E110°59'21"	N35°36'37"	0.23076±0.00201	0.87	2X	451.37
38	YCMZ	Yuci,Shanxi	E112°42'52"	N37°42'14"	0.21951±0.00099	0.45	2X	429.37
39	LXSEZ	Linxian,Shanxi	E110°29′40"	N37°57'25"	0.22238±0.00701	3.15	2X	434.98
40	LXDSZ	Linxian,Shanxi	E110°29′40"	N37°57'25"	0.22215±0.00100	0.45	2X	434.52
41	LXHZ	Linxian,Shanxi	E110°29′40"	N37°57'25"	0.22509±0.00601	2.67	2X	440.28

42	LYHDZ	Linxian,Shanxi	E110°29′40"	N37°57'25"	0.20962±0.00499	2.38	2X	410.01
43	LYDCGZ	Linyi,Shanxi	E110°46'49"	N35°8'58"	0.21743±0.00600	2.76	2X	425.29
44	LFSZ	Loufan,Shanxi	E111°48'12"	N38°4'23"	0.22587±0.00400	1.77	2X	441.8
45	YCDBZ	Yanchuan,Shaanxi	E110°12'1"	N36°53'2"	0.21732±0.00600	2.76	2X	425.07
46	YCBZ	Yanchuan,Shaanxi	E110°12'1"	N36°53'2"	0.21710±0.00399	1.84	2X	424.64
47	LYLZ	Linyi,Shanxi	E110°46'49"	N35°8'58"	0.24411±0.00801	3.28	2X	477.47
48	LYLJZ	Linyi,Shanxi	E110°46'49"	N35°8'58"	0.22428±0.01000	4.46	2X	438.7
49	BDXZ	Baode,Shanxi	E111°5'33"	N39°1'42"	0.24024±0.00702	2.92	2X	469.91
50	BDYZ	Baode,Shanxi	E111°5'33"	N39°1'42"	0.25294±0.00799	3.16	2X	494.76
51	ZYMZ	Zhongyang,Shanxi	E111°11'8"	N37°21'46"	0.23549±0.00301	1.28	2X	460.62
52	ZYTZ	Zhongyang,Shanxi	E111°11'8"	N37°21'46"	0.22117±0.00599	2.71	2X	432.6
53	QXYZ	Qingxu,Shanxi	E112°21'52"	N37°36'47"	0.22163±0.00898	4.05	2X	433.51
54	XFYZ	Xiangfen,Shanxi	E111°26'54"	N35°52'55"	0.23170±0.00500	2.16	2X	453.21
55	YCPPZ	Yuncheng,Shanxi	E111°0'48"	N35°1'57"	0.24756±0.00300	1.21	2X	484.23
56	JCJZ	Jiaocheng,Shanxi	E112°9'45"	N37°33'27"	0.22359±0.00300	1.34	2X	437.35
57	YJHMZ	Yongji,Shanxi	E110°27'16"	N34°52'22"	0.28574±0.00200	0.7	2X	558.91
58	XFGTZ	Xiangfen,Shaanxi	E111°26'54"	N35°52'55"	0.23253±0.00400	1.72	2X	454.82
59	XFMZ	Xiangfen,Shaanxi	E111°26'54"	N35°52'55"	0.23171±0.00899	3.88	2X	453.22
60	JSLGZ	Jishan,Shanxi	E110°59'21"	N35°36'37"	0.23620±0.01101	4.66	2X	462
61	LXYJZ-1	Linxian,Shanxi	E110°29′40"	N37°57'25"	0.25313±0.00101	0.4	2X	495.13
62	PLTTZ	Pinglu,Shanxi	E111°13'24"	N34°50'34"	0.22847±0.00400	1.75	2X	446.88
63	PLJZ	Pinglu,Shanxi	E111°13'24"	N34°50'34"	0.22810±0.00500	2.19	2X	446.17
64	XFYZZ	Xiangfen,Shanxi	E111°26'54"	N35°52'55"	0.22889±0.00199	0.87	2X	447.71
65	XXYCZ	Xiaxian,Shanxi	E111°13'35"	N35°8'50"	0.23150±0.00299	1.29	2X	452.82
66	XXZYZ	Xiaxian,Shanxi	E111°13'35"	N35°8'50"	0.22883±0.00199	0.87	2X	447.59
67	JCDZ	Jiaocheng,Shanxi	E112°9'45"	N37°33'27"	0.23151±0.00199	0.86	2X	452.83
68	YCDYZ	Yanchuan,Shaanxi	E110°12'1"	N36°53'2"	0.22247±0.00501	2.25	2X	435.16
69	DLXDD	Dali,Shaanxi	E109°56'54"	N34°48'6"	0.22009±0.00099	0.45	2X	430.5
70	DLMYZ	Dali,Shaanxi	E109°56'54"	N34°48'6"	0.23982±0.00199	0.83	2X	469.09
71	DLJDZ	Dali,Shaanxi	E109°56'54"	N34°48'6"	0.22286±0.00100	0.45	2X	435.92
72	PCMZ	Pucheng,Shaanxi	E109°35'32"	N34°57'40"	0.22143±0.00301	1.36	2X	433.12
73	PCYLZ	Pucheng,Shaanxi	E109°35'32"	N34°57'40"	0.21607±0.00099	0.46	2X	422.64
74	PCDDZ	Pucheng,Shaanxi	E109°35'32"	N34°57'40"	0.21774±0.00398	1.83	2X	425.89
75	DLLZ	Dali,Shaanxi	E109°56'54"	N34°48'6"	0.22471±0.00400	1.78	2X	439.54
76	DLLLZ	Dali,Shaanxi	E109°56'54"	N34°48'6"	0.21958±0.00698	3.18	2X	429.49
77	LTGLZ	Lintong,Shaanxi	E109°13'13"	N34°22'21"	0.21910±0.00401	1.83	2X	428.56
78	DLYZ	Dali,Shaanxi	E109°56'54"	N34°48'6"	0.21363±0.00199	0.93	2X	417.87
79	DLGWB	Dali,Shaanxi	E109°56'54"	N34°48'6"	0.21132±0.00099	0.47	2X	413.35
80	YCTZ	Yanchuan,Shaanxi	E110°12'1"	N36°53'2"	0.21351±0.00199	0.93	2X	417.63
81	DLPCZ	Dali,Shaanxi	E109°56'54"	N34°48'6"	0.21503±0.00301	1.4	2X	420.59
82	DLZZ	Dali,Shaanxi	E109°56'54"	N34°48'6"	0.21527±0.00101	0.47	2X	421.06
83	DLFMG	Dali,Shaanxi	E109°56'54"	N34°48'6"	0.21953±0.00400	1.82	2X	429.41
84	DLGDZ	Dali,Shaanxi	E109°56'54"	N34°48'6"	0.21217±0.00301	1.42	2X	415
85	DLBSH	Dali,Shaanxi	E109°56'54"	N34°48'6"	0.21790±0.00100	0.46	2X	426.22

86	DLMZ	Dali,Shaanxi	E109°56'54"	N34°48'6"	0.21693±0.00399	1.84	2X	424.31
87	DLZCBZ	Dali,Shaanxi	E109°56'54"	N34°48'6"	0.22436±0.00101	0.45	2X	438.85
88	PYKDZ	Pingyao,shanxi	E112°10'56"	N37°11'44"	0.21690±0.00399	1.84	2X	424.26
89	PYDZ	Pingyao,shanxi	E112°10'56"	N37°11'44"	0.21525±0.00502	2.33	2X	421.03
90	LXXZZ-2	Linxian,Shanxi	E110°29′40"	N37°57'25"	0.24856±0.00897	3.61	2X	486.18
91	LXYJZ-3	Linxian,Shanxi	E110°29′40"	N37°57'25"	0.23711±0.00901	3.8	2X	463.79
92	LXTHDMZ-1	Linxian,Shanxi	E110°29′40"	N37°57'25"	0.24027±0.00199	0.83	2X	469.97
93	LXSJLNNZ	Linxian,Shanxi	E110°29′40"	N37°57'25"	0.22777±0.00100	0.44	2X	445.52
94	LXCNNZ	Linxian,Shanxi	E110°29′40"	N37°57'25"	0.22209±0.01201	5.41	2X	434.4
95	LXEBCSZ	Linxian,Shanxi	E110°29′40"	N37°57'25"	0.23380±0.00198	0.85	3X	685.98
96	LFCZ	Loufan,shanxi	E111°48'12"	N38°4'23"	0.22388±0.00401	1.79	2X	437.9
97	XFHLZ	Xiangfen,Shanxi	E111°26'54"	N35°52'55"	0.22141±0.00100	0.45	2X	433.07
98	XXDSZ	Xixian,shanxi	E110°56'51"	N36°41'56"	0.20824±0.00200	0.96	2X	407.32
99	LXQKKZ	Linxian,Shanxi	E110°29′40"	N37°57'25"	0.21333±0.00501	2.35	2X	417.27
100	LXMZ	Linxian,Shanxi	E110°29′40"	N37°57'25"	0.21419±0.00300	1.4	2X	418.95
101	LXWWZ	Linxian,Shanxi	E110°29′40"	N37°57'25"	0.21164±0.00999	4.72	2X	413.96
102	LXHGZ	Linxian,Shanxi	E110°29′40"	N37°57'25"	0.21552±0.00300	1.39	2X	421.55
103	LXGJJZ	Linxian,Shanxi	E110°29′40"	N37°57'25"	0.23797±0.00100	0.42	2X	465.47
104	LXXCZ	Linxian,Shanxi	E110°29′40"	N37°57'25"	0.23099±0.00400	1.73	2X	451.82
105	JSNYDBZ	Jishan,Shanxi	E110°59'21"	N35°36'37"	0.22062±0.00132	0.6	3X	647.31
106	JSNYXBZ	Jishan,Shanxi	E110°59'21"	N35°36'37"	0.23332±0.00700	3	2X	456.38
107	JSMHZ	Jishan,Shanxi	E110°59'21"	N35°36'37"	0.20880±0.00100	0.48	2X	408.42
108	JSLYPCZ	Jishan,Shanxi	E110°59'21"	N35°36'37"	0.21158±0.00300	1.42	2X	413.85
109	JSLYYZ	Jishan,Shanxi	E110°59'21"	N35°36'37"	0.20477±0.00600	2.93	2X	400.53
110	JSLYJXZ	Jishan,Shanxi	E110°59'21"	N35°36'37"	0.21033±0.00801	3.81	2X	411.4
111	JSLYMTZ	Jishan,Shanxi	E110°59'21"	N35°36'37"	0.21268±0.00200	0.94	2X	416
112	PLWWZ	Pinglu,Shanxi	E111°13'24"	N34°50'34"	0.22421±0.00200	0.89	2X	438.56
113	PLGDZ	Pinglu,Shanxi	E111°13'24"	N34°50'34"	0.21949±0.00301	1.37	2X	429.33
114	PYLJZ	Pingyao,Shanxi	E112°10'56"	N37°11'44"	0.21049±0.00400	1.9	2X	411.71
115	PYSBL	Pingyao,Shanxi	E112°10'56"	N37°11'44"	0.21092±0.00099	0.47	2X	412.56
116	TGNTSMZ	Taigu,Shanxi	E112°33'26"	N37°25'38"	0.20772±0.00100	0.48	2X	406.31
117	TGFCCZ	Taigu,Shanxi	E112°33'26"	N37°25'38"	0.20773±0.00700	3.37	2X	406.32
118	TGFCJZZ	Taigu,Shanxi	E112°33'26"	N37°25'38"	0.21407±0.00599	2.8	2X	418.72
119	YCNNZ	Yanchuan,Shaanxi	E110°12'1"	N36°53'2"	0.24728±0.00099	0.4	2X	483.68
120	LTMHL	Lintong,Shaanxi	E109°13'13"	N34°22'21"	0.21607±0.00099	0.46	2X	422.63
121	LXQJSTZ	Linxian,Shanxi	E110°29′40"	N37°57'25"	0.21402±0.00101	0.47	2X	418.63
122	YLMTZ	Yanliang,Shaanxi	E109°13'56"	N34°40'6"	0.21094±0.00500	2.37	2X	412.59
123	YCGTZ-1	Yanchuan,Shaanxi	E110°12'1"	N36°53'2"	0.21943±0.00402	1.83	2X	429.21
124	YCGTZ-2	Yanchuan,Shaanxi	E110°12'1"	N36°53'2"	0.21798±0.00100	0.46	2X	426.36
125	YCXCMZ	Yanchuan,Shaanxi	E110°12'1"	N36°53'2"	0.21769±0.00100	0.46	2X	425.81
126	YCTZZ	Yanchuan,Shaanxi	E110°12'1"	N36°53'2"	0.21781±0.00100	0.46	2X	426.04
127	YLCZ	Yanliang,Shaanxi	E109°13'56"	N34°40'6"	0.22868±0.00599	2.62	2X	447.3
128	TGDGHPZ	Taigu,Shanxi	E112°33'26"	N37°25'38"	0.22873±0.00333	1.46	3X	671.1
129	TGYZGZ	Taigu,Shanxi	E112°33'26"	N37°25'38"	0.22545±0.00902	4	2X	440.98

130	YHSZ1	Yonghe,Shanxi	E110°38'18"	N36°45'57"	0.21966±0.00099	0.45	2X	429.66
131	XFGTW	Xiangfen,Shanxi	E111°26'54"	N35°52'55"	0.23414±0.00300	1.28	2X	457.98
132	LXLJZ	Linxian,Shanxi	E110°29′40"	N37°57'25"	0.21962±0.00200	0.91	2X	429.57
133	LXQJSFZ-2	Linxian,Shanxi	E110°29′40"	N37°57'25"	0.21436±0.00701	3.27	2X	419.29
134	QXPGZ	Qingxu,Shanxi	E112°21'52"	N37°36'47"	0.21679±0.00199	0.92	3X	636.06
135	JSDBZ	Jishan,Shanxi	E110°59'21"	N35°36'37"	0.24166±0.00500	2.07	2X	472.69
136	JXCMZ	Jiaxian,Shanxi	E110°59'21"	N35°36'37"	0.23830±0.00500	2.1	2X	466.12
137	TGLZ	Taigu,Shanxi	E112°33'26"	N37°25'38"	0.23076±0.00399	1.73	2X	451.37
138	TGYZ	Taigu,Shanxi	E112°33'26"	N37°25'38"	0.21843±0.00500	2.29	2X	427.25
139	TGMXZ	Taigu,Shanxi	E112°33'26"	N37°25'38"	0.23402±0.00199	0.85	2X	457.75
140	SLCZ	Shilou,Shanxi	E110°50'29"	N37°0'16"	0.21939±0.00401	1.83	2X	429.13
141	SLSZ	Shilou,Shanxi	E110°50'29"	N37°0'16"	0.22792±0.00100	0.44	2X	445.81
142	PLLZ	Pinglu,Shanxi	E111°13'24"	N34°50'34"	0.20949±0.00501	2.39	2X	409.77
143	TGLHZ	Taigu,Shanxi	E112°33'26"	N37°25'38"	0.21327±0.00100	0.47	2X	417.15
144	TGCSZ	Taigu,Shanxi	E112°33'26"	N37°25'38"	0.21982±0.00400	1.82	2X	429.96
145	TGGYH	Taigu,Shanxi	E112°33'26"	N37°25'38"	0.22628±0.00500	2.21	2X	442.6
146	TGDMZ	Taigu,Shanxi	E112°33'26"	N37°25'38"	0.22452±0.00599	2.67	2X	439.17
147	TGMXH	Taigu,Shanxi	E112°33'26"	N37°25'38"	0.22705±0.00500	2.2	2X	444.11
148	YCCZ	Yuncheng,Shanxi	E111°0'48"	N35°1'57"	0.22128±0.00600	2.71	2X	432.83
149	YCHMZ	Yuncheng,Shanxi	E111°0'48"	N35°1'57"	0.21958±0.00099	0.45	2X	429.5
150	YCMZZ	Yuncheng,Shanxi	E111°0'48"	N35°1'57"	0.22926±0.00401	1.75	2X	448.44
151	LYWMZ	Linyi,Shanxi	E110°46'49"	N35°8'58"	0.21778±0.00100	0.46	2X	425.98
152	LYZZLZ	Linyi,Shanxi	E110°46'49"	N35°8'58"	0.21480±0.00200	0.93	2X	420.15
153	XFGT-1	Xiangfen,Shanxi	E111°26'54"	N35°52'55"	0.22732±0.00100	0.44	2X	444.64
154	XFGT-2	Xiangfen,Shanxi	E111°26'54"	N35°52'55"	0.21909±0.00300	1.37	2X	428.54
155	YCCMZZ	Yuci,Shanxi	E112°42'52"	N37°42'14"	0.23047±0.00602	2.61	2X	450.79
156	YCNTZ	Yuci,Shanxi	E112°42'52"	N37°42'14"	0.22199±0.00100	0.45	2X	434.21
157	YCYZ	Yuci,Shanxi	E112°42'52"	N37°42'14"	0.22177±0.00599	2.7	2X	433.79
158	TGLJZ-1	Taigu,Shanxi	E112°33'26"	N37°25'38"	0.22660±0.00499	2.2	2X	443.23
159	TGLJZ-2	Taigu,Shanxi	E112°33'26"	N37°25'38"	0.22505±0.00401	1.78	2X	440.2
160	LMX1	Linxian,Shanxi	E110°29′40"	N37°57'25"	0.22396±0.00199	0.89	2X	438.06
161	LXRHZ	Linxian,Shanxi	E110°29′40"	N37°57'25"	0.22202±0.00799	3.6	2X	434.27
162	LXYZ	Linxian,Shanxi	E110°29′40"	N37°57'25"	0.21734±0.00200	0.92	2X	425.11
163	LXMZ	Linxian,Shanxi	E110°29′40"	N37°57'25"	0.21845±0.00701	3.21	2X	427.29
164	LXSTZ	Linxian,Shanxi	E110°29′40"	N37°57'25"	0.21212±0.00401	1.89	2X	414.9
165	LXHTZ	Linxian,Shanxi	E110°29′40"	N37°57'25"	0.23116±0.00700	3.03	2X	452.14
166	LXDLLZ	Linxian,Shanxi	E110°29′40"	N37°57'25"	0.22425±0.00300	1.34	2X	438.64
167	LXHHZ	Linxian,Shanxi	E110°29′40"	N37°57'25"	0.21893±0.00300	1.37	2X	428.22
168	QXJLCZ	Qixian,Shanxi	E112°20'32"	N37°21'50"	0.22113±0.00599	2.71	2X	432.54
169	LYYZ	Linyi,Shanxi	E110°46'49"	N35°8'58"	0.21753±0.00798	3.67	2X	425.48
170	LYDCYZ	Linyi,Shanxi	E110°46'49"	N35°8'58"	0.22302±0.00201	0.9	2X	436.22
171	XXLYKZ	Xiaxian,Shanxi	E111°13'35"	N35°8'50"	0.22963±0.00200	0.87	2X	449.16
172	JSBZY	Jishan,Shanxi	E110°59'21"	N35°36'37"	0.22605±0.00099	0.44	2X	442.15
173	XFZSGT	Xiangfen,Shanxi	E111°26'54"	N35°52'55"	0.22146±0.00100	0.45	2X	433.18

174	XFWSGT	Xiangfen,Shanxi	E111°26'54"	N35°52'55"	0.23110±0.00601	2.6	2X	452.04
175	TYPZZ	Taiyuan,Shanxi	E112°33'23"	N37°52'37"	0.22522±0.00500	2.22	2X	440.53
176	LXMZLMZ	Linxian,Shanxi	E110°29′40"	N37°57'25"	0.22536±0.00602	2.67	2X	440.8
177	LXMALSZ	Linxian,Shanxi	E110°29′40"	N37°57'25"	0.23322±0.00401	1.72	2X	456.18
178	YCHTZ	Yanchuan,Shaanxi	E110°12'1"	N36°53'2"	0.22174±0.00698	3.15	2X	433.73
179	JXHDDZ	Jiaxian,Shanxi	E110°29'50"	N38°1'32"	0.21477±0.00399	1.86	2X	420.09
180	YLYZ	Yanliang,Shaanxi	E109°13'56"	N34°40'6"	0.21700±0.00200	0.92	2X	424.46
181	YLXZ	Yanliang,Shaanxi	E109°13'56"	N34°40'6"	0.21617±0.00499	2.31	2X	422.82
182	YLLLZ	Yanliang,Shaanxi	E109°13'56"	N34°40'6"	0.21883±0.00400	1.83	2X	428.03
183	PCZDZ	Pucheng,Shaanxi	E109°13'56"	N34°40'6"	0.21688±0.00499	2.3	2X	424.22
184	XADBZ	Xian,Shaanxi	E125°9'19"	N42°55'59"	0.21073±0.00200	0.95	2X	412.18
185	YCBZZ	Yanchuan,Shaanxi	E110°12'1"	N36°53'2"	0.21358±0.00199	0.93	2X	417.77
186	DLNZ	Dali,Shaanxi	E109°56'54"	N34°48'6"	0.22617±0.00301	1.33	2X	442.38

Note: The tested materials were preserved in the National Horticultural Germplasm Repository – Jujube Subrepository by grafting from 2011 to 2015.
