# Supplementary material for: Genomic C-Value Variation Analysis in Jujube (Ziziphus jujuba Mill.) in the Middle Yellow River Basin
Source: Plants (Basel). 2023 Feb 14;12(4):858. doi: 10.3390/plants12040858 (PMC9962250; doi:10.3390/plants12040858)
Supplement: Supplementary file 1 [file plants-12-00858-s001.zip › plants-2037022-supplementary/S1-S4/TableS4.pdf]

TableS4 Analysis of least significant difference (LSDs) of genomic C-values of jujube in Sub-regions

Sub-region	Germplasm name	Genomic C-value
Yonghe	YHTZ	0.23991±0.00499 A
	YHSZ1	0.21966±0.00099 B
	F=37.885, P<0.01	
Loufan	LFCZ	0.22388±0.00401 A
	LFSZ	0.22587±0.00400 A
	F=0.311, P>0.05	
Xiangfen	XFHLZ	0.22141±0.00100 BC
	XFGTW	0.23414±0.00300 A
	XFZSGT	0.22146±0.00100 BC
	XFWSGT	0.23110±0.00601 A
	XFGTZ	0.23253±0.00400 A
	XFMZ	0.23171±0.00899 A
	XFYZZ	0.22889±0.00199 AB
	XFGT-1	0.22732±0.00100 AB
	XFGT-2	0.21909±0.00300 C
	XFYZ	0.23170±0.00500 A
	F=4.399, P<0.01	
Jishan	JSNYDBZ	0.22062±0.00132 CDEF
	JSNYXBZ	0.23332±0.00700 BC
	JSMHZ	0.20880±0.00100 EFG
	JSLYPCZ	0.21158±0.00300 DEFG
	JSLYYZ	0.20477±0.00600 G
	JSLYJXZ	0.21033±0.00801 FG
	JSLYMTZ	0.21268±0.00200 BCDE
	JSSZ	0.22667±0.01000 BCDE
	JSHMZ	0.22205±0.00300 CDEF
	JSZYZ	0.23076±0.00201 BCD
	JSDBZ	0.24166±0.00500 B
	JSBZY	0.22605±0.00099 BCDEF
	JSLGZ	0.23620±0.01101 BC
	JSCZ	0.25719±0.00301 A
	F=8.104, P<0.01	
Pinglu	PLWWZ	0.22421±0.00200 AB
	PLGDZ	0.21949±0.00301 B
	PLLZ	0.20949±0.00501 C
	PLTTZ	0.22847±0.00400 A
	PLJZ	0.22810±0.00500 AB
	F=9.976, P<0.01	
Pingyao	PYLJZ	0.21049±0.00400 b
	PYSBL	0.21092±0.00099 b

	PYBJZ	0.22559±0.00399 a
	PYKDZ	0.21690±0.00399 b
	PYDZ	0.21525±0.00502 b
	F=4.17, P<0.05	
Taigu	TGNTSMZ	0.20772±0.00100 F
	TGFCCZ	0.20773±0.00700 F
	TGFCJZZ	0.21407±0.00599 DF
	TGDGHPZ	0.22873±0.00333 AB
	TGYZGZ	0.22545±0.00902 ABC
	TGLZ	0.23076±0.00399 A
	TGYZ	0.21843±0.00500 CDEF
	TGMXZ	0.23402±0.00199 A
	TGLHZ	0.21327±0.00100 F
	TGCSZ	0.21982±0.00400 BCD
	TGGYH	0.22628±0.00500 ABC
	TGDMZ	0.22452±0.00599 ABC
	TGMXH	0.22705±0.00500 ABC
	TGLJZ-1	0.22660±0.00499 ABC
	TGLJZ-2	0.22505±0.00401 ABC
	F=5.478, P<0.01	
Yuci	YCZCGYZ	0.21036±0.00200 BC
	YCZCTSZ	0.21307±0.01099 BC
	YCZZXSYZ	0.20358±0.00599 C
	YCMZ	0.21951±0.00099 ABC
	YCCMZZ	0.23047±0.00602 A
	YCNTZ	0.22199±0.00100 AB
	YCYZ	0.22177±0.00599 AB
	F=4.039, P<0.05	
Lintong	LTMHL	0.21607±0.00099 A
	LTGLZ	0.21910±0.00401 A
	F=2.001, P>0.05	
Yanchuan	YCNNZ	0.24728±0.00099 A
	YCLYDSZ	0.21008±0.01901
	YCGTZ-1	0.21943±0.00402 BC
	YCGTZ-2	0.21798±0.00100 BC
	YCXCMZ	0.21769±0.00100 BC
	YCTZZ	0.21781±0.00100 BC
	YCCMZ	0.23138±0.00010 AB
	YCDBZ	0.21732±0.00600 B
	YCHTZ	0.22174±0.00698 BC
	YCBZ	0.21710±0.00399
	YCDYZ	0.22247±0.00501

	YCTZ	0.21351±0.00199
	YCBZZ	0.21358±0.00199
	F=3.862, P<0.01	
Yanliang	YLMTZ	0.21094±0.00500 b
	YLCZ	0.22868±0.00599 a
	YLYZ	0.21700±0.00200 b
	YLXZ	0.21617±0.00499 b
	YLLLZ	0.21883±0.00400 b
	F=6.058, P<0.05	
Dali	DLMGX-1	0.22215±0.00500 BCDE
	DLXDD	0.22009±0.00099 BCDEF
	DLMYZ	0.23982±0.00199 A
	DLJDZ	0.22286±0.00100 BCD
	DLLZ	0.22471±0.00400 BC
	DLLLZ	0.21958±0.00698 BCDEF
	DLYZ	0.21363±0.00199 EF
	DLGWB	0.21132±0.00099 F
	DLPCZ	0.21503±0.00301 DEF
	DLZZ	0.21527±0.00101 DEF
	DLFMG	0.21953±0.00400 BCDEF
	DLGDZ	0.21217±0.00301 F
	DLNZ	0.22617±0.00301 B
	DLBSH	0.21790±0.00100 BCDEF
	DLMZ	0.21693±0.00399 CDEF
	DLZCBZ	0.22436±0.00101 BC
	F=7.136, P<0.01	
Qingxu	QXMGZ	0.22404±0.00300 a
	QXPGZ	0.21679±0.00199 a
	QXYZ	0.22163±0.00898 a
	F=1.307, P>0.05	
Shilou	SLCZ	0.21939±0.00401 a
	SLSZ	0.22792±0.00100 b
	F=10.895, P<0.05	
Yuncheng	YCCZ	0.22128±0.00600 C
	YCHMZ	0.21958±0.00099 C
	YCMZZ	0.22926±0.00401 B
	YCPPZ	0.24756±0.00300 A
	F=30.772, P<0.01	
Linyi	LYWMZ	0.21778±0.00100 b
	LYZZLZ	0.21480±0.00200 b
	LYYZ	0.21753±0.00798 b
	LYDCGZ	0.21743±0.00600 b

	LYDCYZ	0.22302±0.00201 b
	LYLZ	0.24411±0.00801 a
	LYLJZ	0.22428±0.01000 b
	F=6.342, P<0.05	
Xiaxian	XXLYKZ	0.22963±0.00200 a
	XXYCZ	0.23150±0.00299 a
	XXZYZ	0.22883±0.00199 a
	F=1.005,P>0.05	
Jiaxian	JXHDDZ	0.21477±0.00399 A
	JXCMZ	0.23830±0.00500 B
	F=35.228,P<0.01	
Baode	BDXZ	0.24024±0.00702 a
	BDYZ	0.25294±0.00799 a
	F=4.185,P>0.02	
Zhongyang	ZYMZ	0.23549±0.00301 a
	ZYTZ	0.22117±0.00599 b
	F=14.173,P<0.05	
Jiaocheng	JCJZ	0.22359±0.00300 a
	JCDZ	0.23151±0.00199 b
	F=18.732,P<0.05	
Pucheng	PCMZ	0.22143±0.00301 a
	PCYLZ	0.21607±0.00099 a
	PCDDZ	0.21774±0.00398 a
	PCZDZ	0.21688±0.00499 a
	F=1.092,P>0.05	
Linxian	LXMJW-1	0.25559±0.00455 A
	LXMJW-2	0.24662±0.00335 ABCDE
	LXMJW-3	0.24119±0.0097 ABCDEFG
	LXMJW-4	0.25135±0.00137 AB
	LXXZZ-2	0.24856±0.0085 ABCD
	LXXZZ-1	0.25082±0.00826 AB
	LXWJZDZ	0.24969±0.01293 ABC
	LXYJZ-1	0.25313±0.00008 AB
	LXYJZ-2	0.2378±0.00069 ABCDEFGHI
	LXYJZ-3	0.23711±0.00918 BCDEFGHIJ
	LXTHDMZ-1	0.24027±0.00215 ABCDEFGH
	LXWJZMYZ	0.2273±0.00185 GHIJKLMNO
	LXSJLNNZ	0.22777±0.00141 FGHIJKLMNO
	LXQYNNZ	0.21633±0.00311 KLMNOP
	LXCNNZ	0.22209±0.0121 HIJKLMNOP
	LXXZZ-2	0.21841±0.0074 KLMNOP
	LXYZCZ	0.21886±0.00524 JKLMNOP

	LXKYDSZ	0.23908±0.0003 ABCDEFGH
	LXEBCSZ	0.2338±0.00265 BCDEFGHIJK
	LXEBCCMZ	0.23048±0.00431 DEFGHIJKLM
	LXLJHPBB	0.24204±0.00591 ABCDEFG
	SXLXZ22	0.22982±0.0068 EFGHIJKLMN
	SXLXZ23	0.2122±0.00666 MNOP
	SXLXZ24	0.2144±0.0007 KLMNOP
	SXLXZ25	0.21333±0.00527 LMNOP
	SXLXZ26	0.21418±0.00299 LMNOP
	LXWWZ	0.21163±0.01031 NOP
	LXYLLZ	0.20582±0.00153 P
	LXLJPAAZ	0.24571±0.00771 ABCDEF
	LXHGZ	0.21552±0.00298 KLMNOP
	LXGJJZ	0.23797±0.00104 ABCDEFGH
	LXXCZ	0.23099±0.00449 DEFGHIJKL
	LXQJSTZ	0.21403±0.00069 LMNOP
	LXYJMYZ	0.23151±0.00345 CDEFGHIJKL
	LXLJZ	0.21962±0.00232 IJKLMNOP
	LXQJSLJZ	0.21703±0.00684 KLMNOP
	LXQJSLJZ	0.21935±0.00071 JKLMNOP
	LXQJSDMZ	0.22379±0.00643 GHIJKLMNOP
	LXQJSFZ-2	0.21436±0.00656 LMNOP
	LMX1	0.22396±0.00239 GHIJKLMNOP
	LXSEZ	0.22238±0.00664 HIJKLMNOP
	LXRHZ	0.22202±0.00831 HIJKLMNOP
	LXYZ	0.21734±0.00234 KLMNOP
	LXMZ	0.21845±0.00663 KLMNOP
	LXDSZ	0.22215±0.0004 HIJKLMNOP
	LXSTZ	0.21212±0.00365 NOP
	LXHTZ	0.23116±0.00672 DEFGHIJKL
	LXHZ	0.22509±0.0064 GHIJKLMNO
	LXDLLZ	0.22425±0.00325 GHIJKLMNO
	LXHHZ	0.21893±0.00281 JKLMNOP
	LYHDZ	0.20962±0.00484 OP
	LXMZLMZ	0.22536±0.00646 GHIJKLMNO
	LXMALSZ	0.23322±0.00405 BCDEFGHIJK
	F = 14.89, p < 0.0001	
Xian	XADBZ	0.21073±0.00200
Xingxian	XXLPZ	0.22463±0.00699
Yongji	YJHMZ	0.28574±0.00200
Taiyuan	TYPZZ	0.22522±0.00500
Qixian	QXJLCZ	0.22113±0.00599

Xixian	XXDSZ	0.20824±0.00200

The data in the table is mean ± standard deviation. Means with the same lowercase letters were not significantly different at ($P < 0.05$). Means with the same capital letters were not significantly different at ($p < 0.01$)
